# Six‐Month Use of Droxidopa for Neurogenic Orthostatic Hypotension

**DOI:** 10.1002/mdc3.12726

**Published:** 2019-03-07

**Authors:** Clément François, Cyndya A. Shibao, Italo Biaggioni, Amy M. Duhig, Kim McLeod, Augustina Ogbonnaya, Apryl Quillen, Joan Cannon, Byron Padilla, Binglin Yue, Laurie Orloski, Steven M. Kymes

**Affiliations:** ^1^ Lundbeck Deerfield IL USA; ^2^ Vanderbilt University Medical Center Nashville TN USA; ^3^ Xcenda®, LLC Palm Harbor FL USA; ^4^ Pharmite Douglassville PA USA

**Keywords:** droxidopa, falls, neurogenic orthostatic hypotension, prospective study, quality of life

## Abstract

**Background:**

Droxidopa is approved for adult patients with symptomatic neurogenic orthostatic hypotension (nOH); there is limited information regarding effects on symptoms, outcomes, and quality of life (QOL) beyond two weeks of treatment.

**Objective:**

Examine the real‐world experience of patients taking droxidopa after six months of treatment.

**Methods:**

This non‐interventional, US‐based, prospective cohort study utilized a pharmacy hub, identifying patients who recently started droxidopa for nOH treatment. Questionnaires for fall frequency and other patient‐reported outcomes (PROs) were completed at baseline and one, three, and six months following droxidopa initiation.

**Results:**

179 enrolled patients completed baseline surveys. Droxidopa continuation rates were high at months one, three, and six (87%, 79%, and 75%, respectively). From baseline to month one, there was significant reduction in the proportion of patients reporting falling at least once (54.1% vs. 43.0%; *P* = 0.0039), with similar observations at month three (52.9% vs. 44.5%; *P* = 0.0588) and month six (51.4% vs. 40.0%; *P* = 0.0339). Significant improvements from baseline to month one were observed and maintained at months three and six for most PROs, including the Orthostatic Hypotension Symptom Assessment Item 1, Short Falls Efficacy Scale‐International, Sheehan Disability Scale, Physical Component of the 8‐item Short‐Form Health Survey, and Patient Health Questionnaire‐9.

**Conclusions:**

In this non‐interventional prospective study, fewer nOH patients reported falling after one, three, and six months of droxidopa treatment. Further, improvements reported in nOH symptoms, physical function, and QOL measures were maintained for six months following treatment initiation. Results from randomized clinical trials are required to validate the findings.

## Introduction

Neurogenic orthostatic hypotension (nOH) is defined as a sustained orthostatic fall in systolic blood pressure of ≥20 mm Hg or diastolic pressure of ≥10 mm Hg within three minutes of standing up in patients with neurodegenerative and congenital neurological disorders, including Parkinson's disease (PD), multiple system atrophy (MSA), pure autonomic failure (PAF), dopamine beta‐hydroxylase (DβH) deficiency, or nondiabetic autonomic neuropathy (NDAN).^1^, ^2^, ^3^, ^4^ Patients with nOH often present with orthostatic intolerance and recurrent falls.^2^ Falling is an important risk factor for hip fracture and head trauma,^2^ and fear of falling can have both physical and psychosocial implications.^5^, ^6^ Recent data show that a higher propensity of falls among patients with nOH and PD (relative to patients with PD alone) leads to increased healthcare utilization and costs.^7^

The primary goal of treatment in symptomatic nOH is to reduce symptoms without inducing unacceptable side effects.^1^, ^2^, ^8^, ^9^, ^10^ Pharmacologic agents for nOH treatment include non–Food and Drug Administration (FDA)‐approved agents (such as fludrocortisone and midodrine) and FDA‐approved droxidopa.^10^, ^11^ Droxidopa was approved in 2014, with an orphan drug indication for the treatment of orthostatic intolerance symptoms in patients with symptomatic nOH caused by primary autonomic failure (PD, MSA, and PAF), DβH deficiency, and NDAN. Overall, placebo‐controlled trials have demonstrated improvements in nOH symptoms with droxidopa treatment over one to two weeks and a reduction in the rate of falls, with post hoc evidence of a reduced fear of falling.^12^, ^13^, ^14^, ^15^, ^16^

Few studies have described the burden of nOH, associated disease states, quality of life (QOL),^17^, ^18^ and comorbidities^19^, ^20^, ^21^, ^22^, ^23^; Thus far, no studies have prospectively investigated the impact of nOH on falls, fear of falling, QOL, functioning, depression, or the impact of treatment of nOH on those outcomes. The approval of droxidopa provides an opportunity for a prospective study of its impact on falling behavior and patient‐reported outcomes (PROs) in a “real‐world longitudinal setting.” This study, utilizing a central NORTHERA® pharmacy hub (HUB) by Lash Group for patient identification, was designed to examine the experience of patients newly prescribed droxidopa for the treatment of nOH for up to six months. Among the outcomes assessed in this study, of key interest was the effect of droxidopa initiation on the self‐reported incidence of falls, along with the persistence of this and other effects (i.e., on orthostatic symptoms and QOL) with droxidopa treatment over six months.

## Methods

### Study Design

This was a non‐interventional, US‐based, prospective cohort study in patients newly initiating droxidopa for the treatment of nOH. Data for this study were reported by study participants and the HUB, and collected using case report forms. Enrolled patients provided verbal consent, and completed the online, paper, or telephone interview assessment at five time points during the six‐month follow‐up (screening, baseline, and months 1, 3, and 6).

For study approval, the protocol and informed consent form were submitted to Schulman Institutional Review Board, a central institutional review board. This study was conducted in accordance with the protocol and was consistent with the International Council for Harmonisation Standards of Good Clinical Practice and the applicable regulatory requirements. Details of the study design are in the Supporting document.

### Participant Selection

Eligible patients included adults aged ≥18 years, with a diagnosis of an underlying disorder of primary autonomic failure (PD, MSA, PAF), DβH deficiency, or NDAN, and who were enrolled in the HUB and were newly initiating treatment with droxidopa (no treatment in the prior year). Patients were required to have a diagnosis of nOH, as determined by the physician making the treatment referral. Eligible nOH patients prescribed droxidopa (irrespective of the underlying disorder) were enrolled in the HUB. Patients were required to speak and understand verbal and written instructions in English.

Exclusion criteria included prior enrollment in this study; diagnosis of dementia or Alzheimer's disease, schizophrenia, or other psychiatric disorder; and non‐ambulatory or a wheelchair user. Study personnel, their subordinates, or immediate family members were also excluded.

### Study Treatment

The treating physician made the decision to prescribe droxidopa to patients before the patient was referred to the HUB pharmacy and invited to participate in the study. Even if they stopped taking droxidopa, patients who enrolled in the study were retained. Continued participation in the study was not a condition for continuing droxidopa treatment.

### Study Assessments

Demographic and clinical characteristics were collected at baseline (and updated at month one, when applicable) to characterize the study population. Patient demographics included age, sex, race/ethnicity, marital status, living status, education, income, health insurance, and work status; clinical characteristics included comorbidities and medications.

Information on the use of droxidopa, including starting dose, duration of treatment, and rate of and reason for discontinuation of treatment, was collected from specialty pharmacy records and the HUB based on data recorded by nurses or HUB personnel.

The patient falls questionnaire (developed for this study) was used to evaluate the frequency of falls. Patients were asked if they had one or more falls in the past month (e.g., yes or no). All patients reporting a fall were asked how many falls they experienced and if they had a fall that required medical attention. All PRO assessments were completed at baseline and months one, three, and six, and included the Orthostatic Hypotension Symptom Assessment, Item 1 (OHSA‐1),^24^ patient falls questionnaire, Short Falls Efficacy Scale‐International (FES‐I),^25^ Sheehan Disability Scale (SDS),^26^ Physical Component of the 8‐item Short‐Form Health Survey (SF‐8),^27^ and the Patient Health Questionnaire‐9 (PHQ‐9).^28^ Because OHSA‐1 requires patients to average their problems and patients reported that their functioning varied on a daily basis, indicating patient difficulty in rating themselves, a self‐reported question was added to the domain of good days and bad days.

### Data Collection and Monitoring

Data collected from the study assessments were entered into an electronic data capture system managed by the study central intake center, with data from the HUB pharmacy received electronically and merged with the data from the electronic data capture system.

### Lost to Follow‐up

Lost to follow‐up (LTFU) was assessed, and patients were considered LTFU if they did not complete at least one outcome questionnaire at the six‐month follow‐up mark.

### Statistical Analysis

Categorical data were summarized using frequencies and proportions, while continuous data were summarized using means and standard deviations (SDs). For each outcome, results were tabulated for patients who responded to at least one question related to that outcome. The statistical significance of differences between the follow‐up and baseline for fall and PRO measures were determined using paired *t* tests for continuous measures and McNemar's test for binary measures. A univariate generalized estimating equations model with multinomial distributions and an independent covariance matrix was used for categorical measures.

The association between LTFU at month six and select baseline characteristics (age, sex, primary diagnosis, midodrine use, fludrocortisone use, living status, and droxidopa treatment status at month six) and baseline outcomes (fall, OHSA‐1, FES‐I, SDS, SF‐8, and PHQ‐9) were assessed using multivariable logistic regression models. Details of the analyses are available in the Supporting document.

All statistical analyses were conducted using SAS version 9.3, and a two‐sided probability <0.05 was considered statistically significant.

## Results

### Baseline Characteristics

The HUB allowed for the identification of 415 eligible patients who were contacted to participate in the study, and 232 patients were enrolled. The reasons patients were not enrolled were lack of interest (n = 72), no response (n = 69), or meeting an exclusion criterion (n = 42). Of those who met the study inclusion criteria and provided consent, 53 were LTFU before completion of the baseline survey, and 179 patients completed baseline surveys. In the follow‐up period, 140, 121, and 109 patients completed at least one outcome measure at months one, three, and six, respectively.

Baseline demographic and clinical data (Tables 1 and 2) were available for 179 patients. Mean age was 62.8 years. The most frequent diagnoses were autonomic failure without identifiable cause (65.4%) and PD (33.0%). On average, patients had their first nOH symptom 10 years before and were diagnosed five years before being enrolled in the study. Many patients were taking midodrine (28.5%), fludrocortisone acetate (27.9%), and levodopa/carbidopa (24.0%) during enrollment (Table 2).

**Table 1:** Baseline demographics

	All patients	
Characteristic	n = 179	
Age in years, mean (SD)	62.8	(17.4)
Sex, n (%)		
Female	92	(51.4%)
Male	82	(45.8%)
Unknown	5	(2.8%)
Race, n (%)		
Black	9	(5.0%)
Hispanic	5	(2.8%)
Non‐hispanic white	153	(85.5%)
Other	7	(4.0%)
Unknown	5	(2.8%)
Marital status, n (%)		
Married/cohabiting	132	(73.7%)
Single (divorced, never married/not cohabitating, widowed)	46	(25.7%)
Unknown	1	(0.6%)
Living status, n (%)		
Alone	21	(11.7%)
Long‐term care facility	1	(0.6%)
Living with spouse/significant other	137	(76.5%)
Living with someone other than spouse/significant other	16	(8.9%)
Other	3	(1.7%)
Unknown	1	(0.6%)
Education status, n (%)		
College or university degree (2 or 4‐year degree)	52	(29.1%)
Graduate degree	34	(19.0%)
High school diploma (or GED) or less	33	(18.4%)
Some college or certificate program	58	(32.4%)
Unknown	2	(1.1%)
Income, n (%)		
$0–$35,000	50	(27.9%)
$35,001–$50,000	32	(17.9%)
$50,001–$75,000	21	(11.7%)
$75,001–$100,000	22	(12.3%)
>$100,000	17	(9.5%)
Prefer not to answer	28	(15.6%)
Unknown	9	(5.0%)
Health insurance, n (%)		
No insurance	2	(1.1%)
Insurance through employer	46	(25.7%)
Insurance through private carrier	13	(7.3%)
Insurance through Medicaid	19	(10.6%)
Insurance through Medicare	104	(58.1%)
Work status, n (%)		
Employed (part‐time or full‐time)	32	(17.9%)
Homemaker	10	(5.6%)
On disability	31	(17.3%)
Retired	92	(51.4%)
Student	2	(1.1%)
Unemployed	9	(5.0%)
Unknown	3	(1.7%)

Abbreviations: GED, general equivalency diploma; SD, standard deviation.

**Table 2:** Baseline nOH diagnosis characteristics and clinical characteristics

	All patients	
Characteristic	n = 179	
nOH diagnosis characteristics		
Primary diagnosis, n (%)		
Non‐diabetic autonomic neuropathy	2	(1.1%)
Parkinson's disease	59	(33.0%)
Autonomic failure without identifiable cause	117	(65.4%)
Unknown*	1	(0.5%)
Years to first symptom, mean (SD)	10	11.4
Years to diagnosis, mean (SD)	5	7.4
Treating physician, n (%)		
Cardiologist	129	(72.1%)
Pulmonologist	24	(13.4%)
Urologist	35	(19.6%)
Neurologist	124	(69.3%)
Endocrinologist	19	(10.6%)
Clinical characteristics		
Medication usage in >5% of patients,† n (%)		
Midodrine (Orvaten, ProAmatine)	51	(28.5%)
Fludrocortisone acetate	50	(27.9%)
Levodopa‐carbidopa (Sinemet)	43	(24.0%)
Pyridostigmine (Mestinon)	13	(7.3%)
Dopamine agonists	12	(6.7%)
Pseudoephedrine or ephedrine	12	(6.7%)
Monoamine oxidase B inhibitors	11	(6.1%)
Carbidopa/levodopa/entacapone (Stalevo)	10	(5.6%)
Medical histories in >5% of patients,‡ n (%)		
Arthritis	57	(31.8%)
Depression	48	(26.8%)
Heart disease	35	(19.6%)
High blood pressure	30	(16.8%)
Asthma	25	(14.0%)
Chronic obstructive pulmonary disease	16	(8.9%)
Diabetes	15	(8.4%)
Stroke	12	(6.7%)
Obesity	11	(6.1%)
Cancer	10	(5.6%)
Urinary tract infection	9	(5.0%)

Abbreviations: AIDS, acquired immunodeficiency syndrome; COMT, catechol‐O‐methyl transferase; HIV, human immunodeficiency virus; nOH, neurogenic orthostatic hypotension; SD, standard deviation.

* No underlying cause of nOH was reported for these patients.

† Agents used by <5% of patients included amantadine (Symmetrel; 4.5%), levodopa (Larodopa or Dopar; 3.9%), anticholinergics (2.2%), COMT inhibitors (1.7%), carbidopa (Lodosyn; 1.1%), desmopressin acetate (1.1%), erythropoietin (Epogen; 0.6%), and apomorphine (Apokyn; 0.6%).

‡ Medical histories in <5% of patients included ataxia (2.8%), kidney failure (1.1%), liver disease (1.1%), and HIV/AIDS (0%).

### Droxidopa Dose and Duration

The mean (SD) dose for droxidopa (based on the first prescription) was 1,014.5 (447.6) mg daily, with a mean (SD) treatment duration of 20.2 (11.2) days in the first month. At month one, 122 patients (87.1%) remained on treatment, 13 patients (9.3%) discontinued treatment, and five (3.6%) never initiated treatment. Reasons for discontinuation were adverse events (n = 4), patient choice (n = 2), treatment with alternative medication (n = 1), and other/unknown (n = 6). Treatment continuation rates among those responding to at least one outcome measure at months three and six were 79.3% (96/121) and 75.2% (82/109), respectively. The most common reasons for discontinuation after month one were patient or prescriber choice (n = 6 at both time points), adverse events (n = 3 at month three; n = 6 at month six), and other/unknown (n = 11 and n = 12, respectively).

### Falls

Months one, three, and six fall data were available for 135, 119, and 105 patients, respectively (Table 3). There was a reduction in the proportion of patients who reported having at least one fall from baseline to month one (54.1% vs. 43.0%; *P* < 0.01). Among the patients who fell at least once, the number of falls experienced during the previous month was not significantly different from baseline. The mean number of falls requiring medical services was not significantly different from baseline at any of the follow‐up visits. The reduction in the proportion of patients having at least one fall approached but did not reach statistical significance at the month three assessment; however, statistical significance was reached at month six (Table 3).

**Table 3:** Fall rates

	All patients contributing month 1 data					All patients contributing month 3 data					All patients contributing month 6 data				
	Baseline		1 month			Baseline		3 months			Baseline		6 months		
Characteristic	n = 135		n = 135		*P‐*value	n = 119		n = 119		*P‐*value	n = 105		n = 105		*P*‐value
Fall in the past month, n (%)															
Yes	73	(54.1%)	58	(43.0%)	<0.01	63	(52.9%)	53	(44.5%)	0.06	54	(51.4%)	42	(40.0%)	0.03
No	62	(45.9%)	77	(57.0%)		56	(47.1%)	66	(55.5%)		51	(48.6%)	63	(60.0%)	
Number of falls in the past month, n (%)															
0	62	(45.9%)	77	(57.0%)	0.27	56	(47.1%)	66	(55.5%)	0.48	51	(48.6%)	63	(60.0%)	0.11
1	14	(10.4%)	15	(11.1%)		14	(11.8%)	12	(10.1%)		13	(12.4%)	9	(8.6%)	
2	17	(12.6%)	9	(6.7%)		14	(11.8%)	13	(10.9%)		11	(10.5%)	18	(17.1%)	
3	11	(8.1%)	12	(8.9%)		9	(7.6%)	11	(9.2%)		6	(5.7%)	5	(4.8%)	
4	8	(5.9%)	5	(3.7%)		7	(5.9%)	5	(4.2%)		7	(6.7%)	1	(1.0%)	
5	6	(4.4%)	12	(8.9%)		6	(5.0%)	3	(2.5%)		5	(4.8%)	3	(2.9%)	
6–10	11	(8.1%)	3	(2.2%)		7	(5.9%)	6	(5.0%)		6	(5.7%)	3	(2.9%)	
>10	6	(4.4%)	2	(1.5%)		6	(5.0%)	3	(2.5%)		6	(5.7%)	3	(2.9%)	
Fall required medical services, n (%)															
Yes	16	(21.9%)	12	(20.7%)	0.37	13	(20.6%)	10	(18.9%)	0.7389	12	(22.2%)	9	(21.4%)	1.00
No	57	(78.1%)	44	(75.9%)		50	(79.4%)	43	(81.1%)		42	(77.8%)	31	(73.8%)	
Missing	0	(0.0%)	2	(3.4%)		0	(0.0%)	0	(0.0%)		0	(0.0%)	2	(4.8%)	
Number of falls requiring medical services, mean (SD)	1.2	5.1	0.8	3.6	0.34	1.3	5.4	0.6	2.6	0.2354	1.4	5.6	0.9	4.5	0.88
Change from baseline in the number of falls requiring medical services, mean (SD)	‐	‐	‐0.4	4.5	‐	‐	‐	‐0.8	5.7	‐	‐	‐	‐0.4	6.8	‐

Abbreviations: SD, standard deviation.

### Patient‐Reported Outcomes

Scores for OHSA‐1 were available for 133, 119, and 102 patients at months one, three, and six, respectively. Mean (SD) scores were 5.7 (2.7) at baseline and 4.3 (2.6) at month one. A statistically significant improvement in the OHSA‐1 score from baseline to month one was observed (mean change from baseline, ‐1.5 [2.8] units; *P* < 0.01), indicating a perceived improvement in the symptoms of nOH. This effect was maintained at months three and six (mean changes from baseline, ‐1.9 [3.0] and ‐2.0 [3.1] units, respectively; both *P* < 0.01).

Data for the FES‐I were available for 126, 112, and 98 patients at months one, three, and six, respectively (Table 4 and Supporting Table S1). A statistically significant reduction from baseline to month one was observed on the global FES‐I scale (mean [SD] change from baseline, ‐1.6 [3.6] units; *P* < 0.01), indicating a significant reduction in fear of falling. SDS data were available for 131, 116, and 103 patients at months one, three, and six, respectively (Supporting Table S2). Statistically significant improvements in function at month one were demonstrated by changes in the SDS global functional impairment score (mean [SD] change from baseline, ‐3.4 [7.2]; *P* < 0.01). At months three and six, statistical significance was maintained for improvement in the global functional impairment score (Supporting Table S2).

**Table 4:** Overall patient concern about falls: Short falls efficacy scale‐international scores

	All patients contributing month 1 data					All patients contributing month 3 data					All patients contributing month 6 data				
	Baseline		1 month			Baseline		3 months			Baseline		6 months		
Characteristic	n = 126		n = 126		*P* value	n = 112		n = 112		*P* value	n = 98		n = 98		*P* value
Overall concern about falling, mean (SD)	17.2	5.0	15.5	4.8	<0.01	16.7	5.2	14.1	4.7	<0.01	16.4	4.9	14.0	5.2	<0.01
Change from baseline in the overall concern about falling, mean (SD)	‐	‐	‐1.6	3.6	‐	‐	‐	‐2.5	4.4	‐	‐	‐	‐2.4	4.5	‐
Overall concern about falling, n (%)															
Low concern (7–8)	7	(5.6%)	10	(7.9%)	<0.01	9	(8.0%)	12	(10.7%)	<0.01	7	(7.1%)	16	(16.3%)	<0.01
Moderate concern (9–13)	23	(18.3%)	34	(27.0%)		23	(20.5%)	39	(34.8%)		21	(21.4%)	32	(32.7%)	
High concern (14–28)	96	(76.2%)	82	(65.1%)		80	(71.4%)	61	(54.5%)		70	(71.4%)	50	(51.0%)	

Abbreviations: SD, standard deviation.

Data for the SF‐8 were available for 115, 107, and 94 patients at months one, three, and six, respectively. Statistically significant improvements from baseline to month one were noted in the mean (SD) Physical Component Summary score (an increase from 33.7 [8.5] to 35.8 [8.6]; *P* < 0.01) and four of the eight individual domains (Supporting Table S3). The Mental Component Summary score significantly improved from baseline only at month six (43.0 [10.4] to 45.2 [11.0]; *P* = 0.0074). Statistically significant improvements from baseline to month one were seen in the reported numbers of good days (n = 130; increase from mean [SD] of 3.1 [2.0] to 4.0 [2.1] days; *P* < 0.0001) and bad days (n = 128; decrease from 3.7 [2.1] to 2.9 [2.0] days; *P* < 0.0001) over the prior week. These improvements in both good and bad days were maintained at months three and six (Supporting Table S4).

Data for the PHQ‐9 were available for 115, 102, and 91 patients at months one, three, and six, respectively (Supporting Table S5). There was a statistically significant reduction from baseline to month one in the mean (SD) global PHQ‐9 score (‐1.3 [4.5]; *P* = 0.0031), indicating reduced depression among participants, with even larger reductions observed at months three (‐1.7 [6.2]; *P* = 0.0093) and six (‐2.6 [5.1]; *P* < 0.0001).

### Lost to Follow‐Up

At month six, 40.8% of patients were LTFU. Having a fall during the baseline period was not associated with LTFU at month six (β: ‐0.325; *P* = 0.340). Additionally, having a fall in the follow‐up period was not associated with LTFU in the subsequent follow‐up period. Similar results were observed for nearly all of the PROs. Baseline OHSA‐1 (β: 0.013; *P* = 0.85), PHQ‐9 (β: ‐0.006; *P* = 0.85), SDS (β: 0.003; *P* = 0.93), SF‐8 Physical Component Summary scores (β: 0.021; *P* = 0.32), and SF‐8 Mental Component Summary scores (β: 0.008; *P* = 0.65) were not significantly associated with LTFU at month six; however, baseline FES‐I (β: 0.101; *P* = 0.04) was associated with LTFU at month six. An analysis comparing the proportions of patients who continued into the subsequent period with those who were LTFU in the subsequent period found no significant differences in the risk of falls between patients continuing and those LTFU in the subsequent treatment period (Table 5). These results indicate that there is a low risk of bias as a result of study discontinuation during any of the treatment periods.

**Table 5:** Lost to follow‐up: Risk of falls

	Risk of falls, %		
Visit	Continuing	Lost to follow‐up	*P*‐value
Baseline	51.4	51.5	0.996
Month 1	42.4	40.9	0.865
Month 3	44.4	41.9	0.810

## Discussion

In this six‐month prospective cohort study of patients initiating droxidopa for treatment of nOH, there was a statistically significant reduction in the proportion of patients who reported having a fall over the first month of therapy, and this reduction in falling was sustained through six months of treatment among those patients reporting results. Improvements were also seen in health‐related QOL, symptoms, and physical function, as documented by improvements in the FES‐I, OHSA‐1, PHQ‐9, SDS, and SF‐8. These improvements were observed at month one and were sustained through month six. A statistically significant improvement was also noted in the number of good and bad days at all three follow‐up visits.

We found a significant 10‐point reduction from baseline to month one in the proportion of patients who reported having a fall (indicating a 20% reduction in risk of falling), with similar reductions in subsequent time periods. In the phase 3 clinical trial (Study NOH306) of droxidopa treatment in patients with PD‐associated nOH, the team evaluated falls as a secondary outcome. The NOH306 team saw a 77% reduction in relative risk for participants receiving droxidopa versus placebo (a reduction in falls per patient‐week from 1.05 in the placebo group vs. 0.4 in the droxidopa group).^15^ It is not surprising that we would see a smaller reduction in falling in a “real‐world” cohort study versus a randomized trial to support drug licensing. In the present cohort study, patient follow‐up was less frequent and fewer steps were taken to ensure patient compliance with therapy and study procedures. Also, there were several differences between the population studied in the HUB study and the clinical trial. The HUB included patients with a wider variety of primary diagnoses, and our HUB study primarily included patients with autonomic failure without an identifiable cause or PD. HUB participants were younger than those in the phase 3 trial in PD^15^ (mean age of 62.8 vs. 72.5 years), and the patients in NOH306 had a greater baseline falling risk. Also, different instruments were used to track falls; participants in NOH306 used an electronic falls calendar, whereas the present study used a fall questionnaire, which relied on a 30‐day recall, and thus, was subject to recall bias.

It is well appreciated that falls are common^29^, ^30^ and rank among the most frequent reasons for hospital admissions in patients with neurologic conditions such as PD,^31^, ^32^, ^33^, ^34^ yet the contribution of nOH to fall risk and the resulting cost of care in this population remains uncertain. The available publications mainly focus on nOH with an underlying PD diagnosis. Pickering et al. conducted a meta‐analysis of six prospective studies of falling in patients with PD, finding that the 90‐day risk of at least one fall was 46%.^30^ A systematic review of 22 studies of falls in patients with PD reported that 60.5% of patients reported at least one fall; and of these patients, 68% experienced recurrent falls.^29^ At baseline, we found that the risk of falling exceeded 50% in the prior month. A US‐based retrospective cohort study reported that patients with PD and nOH have a higher prevalence of pre‐existing comorbidities and a higher rate of medically attended falls than those with PD alone, leading to increased care costs.^7^ Further studies are needed to determine the impact of nOH on the cost of care in a more diverse population.

The high rates of droxidopa continuation at one, three, and six months (87%, 79%, and 75%, respectively) are particularly noteworthy. A prior retrospective cohort study of patients aged ≥50 years with orthostatic hypotension who initiated treatment with fludrocortisone or midodrine (which were the most commonly used prior therapies in the HUB) found that 43% of patients only had one prescription filled during the study period, and that about 30% of patients discontinued or changed their original regimen in the first month of therapy. In that study, median times to discontinuation were 268 days for fludrocortisone and 304 days for midodrine.^35^ Because determining persistence and discontinuation with droxidopa is dependent on a longer follow‐up period, the persistence on treatment in this study was 87% in the first month of therapy, and the rates of continuation over six months were encouraging and suggest a perception of efficacy and tolerability from the patient's perspective.

Although the most common primary diagnosis was autonomic failure without an identifiable cause, study inclusion criteria required all patients to have primary autonomic failure (i.e., PD, MSA, or PAF), DβH deficiency, or NDAN. Therefore, patients were prescribed droxidopa based upon the judgment of their treating physician that they had nOH associated with primary autonomic failure. Many of the common symptoms of nOH are nonspecific; thus, the accurate diagnosis of nOH can be difficult in early forms of autonomic failure and/or without conducting specialized autonomic tests (often only available in specialty autonomic centers). For this reason, we cannot rule out that some of the patients included in the autonomic failure without an identifiable cause diagnosis group presented with symptoms consistent with nOH, but ultimately had other dysautonomia diagnoses such as vasovagal syncope or postural orthostatic tachycardia syndrome; this reflects the experience of what might occur in clinical practice, as would be expected in this “real‐world” study. Nevertheless, in clinical practice, droxidopa should always be prescribed according to the approved labeling for adult patients with symptomatic nOH caused by primary autonomic failure (PD, MSA, PAF), DβH deficiency, or NDAN.

Regarding the limitations of our study, this was an uncontrolled study, and randomized clinical trials need to be conducted to validate these findings. Recruitment and enrollment of patients were conducted by identifying eligible patients via the HUB. Patients who consented and were enrolled in the study may be different from those who did not consent. We relied on patient self‐report for the key outcomes and clinical measures in the study; thus, it is possible that there was recall bias or misclassification. Also, the falls questionnaire used has not been validated; however, the initial question (i.e., have you experienced a fall in the past month?) is based on a similar question (with a six‐month recall period) in a validated instrument for falls assessment, the Fall Risk Questionnaire.^36^

Additionally, although all medication use information was collected at baseline, information on non‐pharmacologic management approaches used by the patients was not collected, which prevented characterization of the potential impact of these symptom management approaches on study outcomes. Furthermore, the lack of a contemporaneous control group reduces the ability to claim causality with treatment. However, given the size of the effects seen in fall reduction and PRO improvement, it is not likely that this is entirely the result of weakness in study design. The study cohort largely included patients with primary diagnoses of autonomic failure and PD; therefore, findings from this study can only be generalized to such patients. Finally, while 40% of the original sample were LTFU, our analyses showed that patients LTFU were similar at baseline for fall behavior and other outcomes as those patients not LTFU. With the exception of fear of falling, this relationship was maintained across all follow‐up periods (fear of falling was associated with LTFU at six months). Thus, it is unlikely that LTFU was a threat to the validity of our study findings for any of our outcomes except for fear of falling.

In conclusion, in this non‐interventional prospective study of nOH patients, fewer patients reported having a fall after one, three, and six months of droxidopa treatment than at baseline. Improvements in nOH symptoms, functionality, and QOL were also reported. Results from randomized clinical trials are required to validate these findings. Furthermore, it is unclear to what extent a “placebo effect” can influence these results, but insight into this question will be gained from additional randomized clinical trial data.

## Author Roles

1. Research Project: A. Conception, B. Organization, C. Execution; 2. Statistical Analysis: A. Design, B. Execution, C. Review and Critique; 3. Manuscript Preparation: A. Writing the First Draft, B. Review and Critique.

C.F.: 1A, 1B, 2A, 2C, 3A, 3B

C.A.S: 2A, 2C, 3A, 3B

I.B.: 2A, 2C, 3A, 3B

A.M.D.: 1A, 1B, 2A, 2C, 3A, 3B

K.M.: 1A, 1B, 2A, 2C, 3A, 3B

A.O.: 2A, 2B, 2C, 3A, 3B

A.Q.: 1A, 1B, 2A, 2C, 3A, 3B

J.C.: 1A, 1B, 2A, 2C, 3A, 3B

B.P.: 1A, 1B, 2A, 2C, 3A, 3B

B.Y.: 2A, 2B, 2C, 3A, 3B

L.O.: 3A, 3B

S.M.K.: 2A, 2C, 3A, 3B

## Disclosures

**Ethical Compliance Statement:** The authors of this manuscript confirm that we have read the Journal's position on issues involved in ethical publication and affirm that this work is consistent with those guidelines. The protocol and informed consent form were submitted to Schulman Institutional Review Board, a central institutional review board, for study approval. This study was conducted in accordance with the protocol and was consistent with the International Council for Harmonisation Standards of Good Clinical Practice and the applicable regulatory requirements.

**Funding Sources and Conflict of Interest:** This study was funded by Lundbeck. Xcenda, LLC received funding from Lundbeck to conduct this study and develop the manuscript. Pharmite received funding from Xcenda for assistance with the manuscript development. Lundbeck provided funding for medical writing and editorial support in the development of this manuscript.

**Financial Disclosures for the previous 12 months:** Clément François was an employee of Lundbeck at the time of the study and is a shareholder of Lundbeck. He is currently an employee of Creativ‐Ceutical. Steven M. Kymes is an employee of Lundbeck and holds stock in Lundbeck. Steven Kymes also held stock in CVS Health and is a co‐author on two patent applications by CVS Health, but has no royalty rights associated with either application. Joan Cannon is an employee of Lundbeck. Byron Padilla was an employee of Lundbeck at the time of the study completion and is now an employee of AbbVie. Cyndya A. Shibao is an employee of Vanderbilt University Medical Center; has grants from the FDA, NIH, and Doris Duke Foundation; and has served as a consultant and on advisory boards for Lundbeck. Italo Biaggioni is an employee of Vanderbilt University Medical Center and has served as a consultant for Theravance Biopharma. Amy M. Duhig is an employee of Xcenda and holds stock in AmerisourceBergen, Abbott, and Eli Lilly and Company. Kim McLeod, Apryl Quillen, Augustina Ogbonnaya, and Binglin Yue are employees of Xcenda, LLC. Laurie Orloski is an employee of Pharmite.

## Supporting information


**Supporting Methods S1.Supporting Table S1.** Patient concern about falls: Short falls efficacy scale‐international scores.
**Supporting Table S2.** Patient disability caused by falls: Sheehan disability scale scores.
**Supporting Table S3.** Quality‐of‐life measure: Short form‐8 health survey.
**Supporting Table S4.** Number of fainting episodes, good days, and bad days.
**Supporting Table S5.** Patient health status: Patient health questionnaire‐9 scores.
