# Supplementary material for: Six‐Month Use of Droxidopa for Neurogenic Orthostatic Hypotension
Source: Mov Disord Clin Pract. 2019 Mar 7;6(3):235–42. doi: 10.1002/mdc3.12726 (PMC6417751; doi:10.1002/mdc3.12726)
Supplement: Supplementary file 1 — Supporting Methods S1.Supporting Table S1. Patient concern about falls: Short falls efficacy scale‐international scores. Supporting Table S2. Patient disability caused by falls: Sheehan disability scale scores. Supporting Table S3. Quality‐of‐life measure: Short form‐8 health survey. Supporting Table S4. Number of fainting episodes, good days, and bad days. Supporting Table S5. Patient health status: Patient health questionnaire‐9 scores. [file MDC3-6-235-s001.docx]

**Supplemental Tables**

**Table S1. Patient Concern About Falls: Short Falls Efficacy Scale-International Scores**

| Characteristic/Individual Items | All Patients Contributing Month 1 Data | | | | | | All Patients Contributing Month 3 Data | | | | | All Patients Contributing Month 6 Data | | | | |
| --- | --- | --- | --- | --- | --- | --- | --- | --- | --- | --- | --- | --- | --- | --- | --- | --- |
|  | Baseline | | | 1 month | | *P*-value | Baseline | | 3 months | | *P*-value | Baseline | | 6 months | | *P*- value |
|  | N | =126 | | N | =126 |  | N | =112 | N | =112 |  | N | =98 | N | =98 |  |
| Getting dressed or undressed, n (%) |  | |  |  |  |  |  |  |  |  |  |  |  |  |  |  |
| Not at all concerned | 37 | | (29.4%) | 47 | (37.3%) | <0.01 | 37 | (33.0%) | 52 | (46.4%) | <0.01 | 33 | (33.7%) | 48 | (49.0%) | 0.04 |
| Somewhat concerned | 59 | | (46.8%) | 64 | (50.8%) |  | 47 | (42.0%) | 45 | (40.2%) |  | 40 | (40.8%) | 33 | (33.7%) |  |
| Fairly concerned | 21 | | (16.7%) | 11 | (8.7%) |  | 19 | (17.0%) | 12 | (10.7%) |  | 17 | (17.3%) | 12 | (12.2%) |  |
| Very concerned | 9 | | (7.1%) | 4 | (3.2%) |  | 9 | (8.0%) | 3 | (2.7%) |  | 8 | (8.2%) | 5 | (5.1%) |  |
| Taking a bath or a shower, n (%) |  | |  |  |  |  |  |  |  |  |  |  |  |  |  |  |
| Not at all concerned | 15 | | (11.9%) | 24 | (19.0%) | 0.02 | 19 | (17.0%) | 31 | (27.7%) | <0.01 | 14 | (14.3%) | 24 | (24.5%) | <0.01 |
| Somewhat concerned | 51 | | (40.5%) | 47 | (37.3%) |  | 44 | (39.3%) | 43 | (38.4%) |  | 43 | (43.9%) | 50 | (51.0%) |  |
| Fairly concerned | 30 | | (23.8%) | 36 | (28.6%) |  | 26 | (23.2%) | 21 | (18.8%) |  | 24 | (24.5%) | 9 | (9.2%) |  |
| Very concerned | 30 | | (23.8%) | 19 | (15.1%) |  | 23 | (20.5%) | 17 | (15.2%) |  | 17 | (17.3%) | 15 | (15.3%) |  |
| Getting in or out of a chair, n (%) |  | |  |  |  |  |  |  |  |  |  |  |  |  |  |  |
| Not at all concerned | 32 | | (25.4%) | 38 | (30.2%) | <0.01 | 34 | (30.4%) | 45 | (40.2%) | 0.06 | 30 | (30.6%) | 44 | (44.9%) | <0.01 |
| Somewhat concerned | 50 | | (39.7%) | 67 | (53.2%) |  | 44 | (39.3%) | 46 | (41.1%) |  | 41 | (41.8%) | 45 | (45.9%) |  |
| Fairly concerned | 30 | | (23.8%) | 18 | (14.3%) |  | 22 | (19.6%) | 17 | (15.2%) |  | 19 | (19.4%) | 7 | (7.1%) |  |
| Very concerned | 14 | | (11.1%) | 3 | (2.4%) |  | 12 | (10.7%) | 4 | (3.6%) |  | 8 | (8.2%) | 2 | (2.0%) |  |
| Unknown | 0 | | (0.0%) | 0 | (0.0%) |  | 0 | (0.0%) | 0 | (0.0%) |  | 0 | (0.0%) | 0 | (0.0%) |  |
| Going up stairs (or down), n (%) |  | |  |  |  |  |  |  |  |  |  |  |  |  |  |  |
| Not at all concerned | 9 | | (7.1%) | 13 | (10.3%) | 0.01 | 10 | (8.9%) | 26 | (23.2%) | <0.01 | 8 | (8.2%) | 24 | (24.5%) | <0.01 |
| Somewhat concerned | 43 | | (34.1%) | 53 | (42.1%) |  | 38 | (33.9%) | 51 | (45.5%) |  | 36 | (36.7%) | 43 | (43.9%) |  |
| Fairly concerned | 42 | | (33.3%) | 34 | (27.0%) |  | 35 | (31.3%) | 23 | (20.5%) |  | 33 | (33.7%) | 15 | (15.3%) |  |
| Very concerned | 32 | | (25.4%) | 26 | (20.6%) |  | 29 | (25.9%) | 12 | (10.7%) |  | 21 | (21.4%) | 16 | (16.3%) |  |
| Unknown | 0 | | (0.0%) | 0 | (0.0%) |  | 0 | (0.0%) | 0 | (0.0%) |  | 0 | (0.0%) | 0 | (0.0%) |  |
| Reaching for something above your head (or on the ground), n (%) |  | |  |  |  |  |  |  |  |  |  |  |  |  |  |  |
| Not at all concerned | 20 | | (15.9%) | 27 | (21.4%) | 0.16 | 20 | (17.9%) | 33 | (29.5%) | <0.01 | 16 | (16.3%) | 28 | (28.6%) | <0.01 |
| Somewhat concerned | 49 | | (38.9%) | 50 | (39.7%) |  | 46 | (41.1%) | 52 | (46.4%) |  | 43 | (43.9%) | 46 | (46.9%) |  |
| Fairly concerned | 35 | | (27.8%) | 24 | (19.0%) |  | 31 | (27.7%) | 18 | (16.1%) |  | 24 | (24.5%) | 14 | (14.3%) |  |
| Very concerned | 22 | | (17.5%) | 25 | (19.8%) |  | 15 | (13.4%) | 9 | (8.0%) |  | 15 | (15.3%) | 10 | (10.2%) |  |
| Unknown | 0 | | (0.0%) | 0 | (0.0%) |  | 0 | (0.0%) | 0 | (0.0%) |  | 0 | (0.0%) | 0 | (0.0%) |  |
| Walking up a slope or (down), n (%) |  | |  |  |  |  |  |  |  |  |  |  |  |  |  |  |
| Not at all concerned | 20 | | (15.9%) | 32 | (25.4%) | 0.03 | 19 | (17.0%) | 24 | (21.4%) | <0.01 | 16 | (16.3%) | 31 | (31.6%) | 0.03 |
| Somewhat concerned | 50 | | (39.7%) | 44 | (34.9%) |  | 43 | (38.4%) | 58 | (51.8%) |  | 40 | (40.8%) | 35 | (35.7%) |  |
| Fairly concerned | 32 | | (25.4%) | 29 | (23.0%) |  | 29 | (25.9%) | 21 | (18.8%) |  | 28 | (28.6%) | 22 | (22.4%) |  |
| Very concerned | 24 | | (19.0%) | 21 | (16.7%) |  | 21 | (18.8%) | 9 | (8.0%) |  | 14 | (14.3%) | 10 | (10.2%) |  |
| Going out to a social event, n (%) |  | |  |  |  |  |  |  |  |  |  |  |  |  |  |  |
| Not at all concerned | 17 | | (13.5%) | 34 | (27.0%) | <0.01 | 19 | (17.0%) | 34 | (30.4%) | <0.01 | 18 | (18.4%) | 32 | (32.7%) | <0.01 |
| Somewhat concerned | 45 | | (35.7%) | 50 | (39.7%) |  | 40 | (35.7%) | 45 | (40.2%) |  | 35 | (35.7%) | 40 | (40.8%) |  |
| Fairly concerned | 32 | | (25.4%) | 24 | (19.0%) |  | 25 | (22.3%) | 20 | (17.9%) |  | 27 | (27.6%) | 12 | (12.2%) |  |
| Very concerned | 32 | | (25.4%) | 18 | (14.3%) |  | 28 | (25.0%) | 13 | (11.6%) |  | 18 | (18.4%) | 14 | (14.3%) |  |
| Unknown | 0 | | (0.0%) | 0 | (0.0%) |  | 0 | (0.0%) | 0 | (0.0%) |  | 0 | (0.0%) | 0 | (0.0%) |  |

**Table S2. Patient Disability Caused by Falls: Sheehan Disability Scale Scores**

| Characteristic | All Patients Contributing Month 1 Data | | | | | All Patients Contributing Month 3 Data | | | | | All Patients Contributing Month 6 Data | | | | |
| --- | --- | --- | --- | --- | --- | --- | --- | --- | --- | --- | --- | --- | --- | --- | --- |
|  | Baseline | | 1 month | | *P*- value | Baseline | | 3 month | | *P*-value | Baseline | | 6 month | | *P*- value |
|  | N | =131 | N | =131 |  | N | =116 | N | =116 |  | N | =103 | N | =103 |  |
|  |  |  |  |  |  |  |  |  |  |  |  |  |  |  |  |
| **Overall Sheehan Disability Scale** |  |  |  |  |  |  |  |  |  |  |  |  |  |  |  |
| N | 56 |  | 56 |  |  | 46 |  |  |  |  | 40 |  |  |  |  |
| Score, mean (SD) | 18.7 | 7.9 | 15.3 | 8.7 | <0.01 | 18 | 8.1 | 14 | 9.5 | <0.01 | 18.5 | 7.7 | 13.4 | 9.7 | <0.01 |
| Change from baseline, mean (SD) | - | - | -3.4 | 7.2 | - | - | - | -5.1 | 6.8 | - | - | - | -4.2 | 7.4 | - |
| **Individual Items** |  |  |  |  |  |  |  |  |  |  |  |  |  |  |  |
| Symptoms have disrupted your work/school |  |  |  |  |  |  |  |  |  |  |  |  |  |  |  |
| N | 56 |  | 56 |  |  | 47 |  |  |  |  | 40 |  |  |  |  |
| Score, mean (SD) | 6.6 | 3.2 | 5.5 | 3.4 | <0.01 | 6 | 3 | 5 | 4.0 | <0.01 | 6.5 | 3.5 | 4.4 | 3.9 | <0.01 |
| Change from baseline, mean (SD) | - | - | -1.1 | 3.1 | - | - | - | -2.2 | 2.7 | - | - | - | -2.0 | 3.1 | - |
| Symptoms have disrupted your social life/leisure activities |  |  |  |  |  |  |  |  |  |  |  |  |  |  |  |
| N | 128 |  | 128 |  |  | 114 |  |  |  |  | 102 |  |  |  |  |
| Score, mean (SD) | 6.2 | 2.8 | 5.3 | 3.0 | <0.01 | 6 | 3 | 5 | 3.0 | <0.01 | 6.0 | 2.9 | 4.6 | 3.2 | <0.01 |
| Change from baseline, mean (SD) | - | - | -1.0 | 2.4 | - | - | - | -1.3 | 2.9 | - | - | - | -1.5 | 3.1 | - |
| Symptoms have disrupted your family life/home responsibilities |  |  |  |  |  |  |  |  |  |  |  |  |  |  |  |
| N | 129 |  | 129 |  |  | 114 |  |  |  |  | 102 |  |  |  |  |
| Score, mean (SD) | 6.2 | 2.8 | 5.2 | 2.9 | <0.01 | 6 | 3 | 5 | 3.2 | <0.01 | 6.2 | 2.6 | 4.4 | 3.3 | <0.001 |
| Change from baseline, mean (SD) | - | - | -1.0 | 2.6 | - | - | - | -1.2 | 3.0 | - | - | - | -1.8 | 3.2 | - |
| Symptoms have disrupted your work/school, n (%) |  |  |  |  |  |  |  |  |  |  |  |  |  |  |  |
| I have not worked/studied at all due to other unrelated disorder | 57 | (43.5%) | 53 | (40.5%) | 0.06 | 54 | (46.6%) | 48 | (41.4%) | 1.00 | 46 | (44.7%) | 48 | (46.6%) | 1.00 |
| Not at all | 7 | (5.3%) | 13 | (9.9%) |  | 8 | (6.9%) | 15 | (12.9%) |  | 7 | (6.8%) | 12 | (11.7%) |  |
| Mildly | 6 | (4.6%) | 13 | (9.9%) |  | 5 | (4.3%) | 16 | (13.8%) |  | 4 | (3.9%) | 18 | (17.5%) |  |
| Moderately | 19 | (14.5%) | 20 | (15.3%) |  | 16 | (13.8%) | 11 | (9.5%) |  | 15 | (14.6%) | 7 | (6.8%) |  |
| Markedly | 18 | (13.7%) | 17 | (13.0%) |  | 15 | (12.9%) | 8 | (6.9%) |  | 13 | (12.6%) | 7 | (6.8%) |  |
| Extremely | 24 | (18.3%) | 15 | (11.5%) |  | 18 | (15.5%) | 18 | (15.5%) |  | 18 | (17.5%) | 11 | (10.7%) |  |
| Symptoms have disrupted your social life/leisure activities, n (%) |  |  |  |  |  |  |  |  |  |  |  |  |  |  |  |
| Unknown | 2 | (0.8%) | 1 | (0.8%) | <0.01 | 2 | (1.7%) | 0 | (0.0%) | 0.26 | 1 | (1.0%) | 0 | (0.0%) | 0.10 |
| Not at all | 8 | (6.1%) | 12 | (9.2%) |  | 10 | (8.6%) | 15 | (12.9%) |  | 7 | (6.8%) | 16 | (15.5%) |  |
| Mildly | 14 | (10.7%) | 26 | (19.8%) |  | 16 | (13.8%) | 30 | (25.9%) |  | 14 | (13.6%) | 27 | (26.2%) |  |
| Moderately | 40 | (30.5%) | 42 | (32.1%) |  | 36 | (31.0%) | 34 | (29.3%) |  | 31 | (30.1%) | 30 | (29.1%) |  |
| Markedly | 46 | (35.1%) | 39 | (29.8%) |  | 34 | (29.3%) | 31 | (26.7%) |  | 35 | (34.0%) | 18 | (17.5%) |  |
| Extremely | 21 | (16.0%) | 11 | (8.4%) |  | 18 | (15.5%) | 6 | (5.2%) |  | 15 | (14.6%) | 12 | (11.7%) |  |
| Symptoms have disrupted your family life/home responsibilities, n (%) |  |  |  |  | <0.01 |  |  |  |  | 0.05 |  |  |  |  | 0.01 |
| Unknown | 1 | (0.8%) | 1 | (0.8%) |  | 1 | (0.9%) | 1 | (0.9%) |  | 1 | (1.0%) | 0 | (0.0%) |  |
| Not at all | 8 | (6.1%) | 9 | (6.9%) |  | 7 | (6.0%) | 16 | (13.8%) |  | 4 | (3.9%) | 16 | (15.5%) |  |
| Mildly | 16 | (12.2%) | 31 | (23.7%) |  | 17 | (14.7%) | 27 | (23.3%) |  | 14 | (13.6%) | 31 | (30.1%) |  |
| Moderately | 36 | (27.5%) | 42 | (32.1%) |  | 31 | (26.7%) | 33 | (28.4%) |  | 27 | (26.2%) | 26 | (25.2%) |  |
| Markedly | 51 | (38.9%) | 38 | (29.0%) |  | 46 | (39.7%) | 29 | (25.0%) |  | 45 | (43.7%) | 19 | (18.4%) |  |
| Extremely | 19 | (14.5%) | 10 | (7.6%) |  | 14 | (12.1%) | 10 | (8.6%) |  | 12 | (11.7%) | 11 | (10.7%) |  |
| Number of days lost (missed school or work or normal activities), mean (SD) | 2.6 | 2.7 | 2.3 | 2.5 | 0.07 | 2.5 | 2.6 | 2.2 | 2.6 | 0.17 | 2.5 | 2.6 | 1.9 | 2.6 | 0.02 |
| Change from baseline, mean (SD) |  |  | -0.3 | 1.8 |  |  |  | -0.4 | 2.5 |  |  |  | -0.6 | 2.6 |  |
| Number of days unproductive, mean (SD) | 2.6 | 2.7 | 2.4 | 2.6 | 0.40 | 2.4 | 2.7 | 2.3 | 2.6 | 0.49 | 2.5 | 2.7 | 1.9 | 2.6 | 0.09 |
| Change from baseline, mean (SD) | - | - | -0.2 | 2.4 | - | - | - | -0.2 | 2.7 | - | - | - | -0.6 | 3.0 | - |

Key: SD – standard deviation.

**Table S3. Quality-of-Life Measure: Short Form-8 Health Survey**

| Characteristic | All Patients Contributing Month 1 Data | | | | | All Patients Contributing Month 3 Data | | | | | All Patients Contributing Month 6 Data | | | | |
| --- | --- | --- | --- | --- | --- | --- | --- | --- | --- | --- | --- | --- | --- | --- | --- |
|  | Baseline | | 1 month | | *P*- value | Baseline | | 3 month | | *P*- value | Baseline | | 6 month | | *P*- value |
|  | N=130 | | N=130 | |  | N=119 | | N=119 | |  | N=102 | | N=102 | |  |
| **Global rating, mean (SD)** |  |  |  |  |  |  |  |  |  |  |  |  |  |  |  |
| N^a^ | 115 |  | 115 |  |  | 107 |  |  |  |  | 94 |  |  |  |  |
| General Health | 37.9 | 6.7 | 40.0 | 7.3 | 0.0020 | 38.0 | 6.9 | 40.6 | 7.8 | 0.0011 | 37.6 | 6.9 | 40.4 | 7.6 | <0.0001 |
| Change from baseline, mean (SD) | - | - | 2.0 | 7.0 |  | - | - | 2.6 | 8.2 |  | - | - | 2.9 | 7.0 |  |
| Physical Functioning | 34.3 | 8.4 | 37.1 | 8.6 | 0.0003 | 34.8 | 8.4 | 37.0 | 9.0 | 0.0096 | 34.0 | 8.4 | 38.2 | 9.5 | <0.0001 |
| Change from baseline, mean (SD) | - | - | 2.8 | 8.1 |  | - | - | 2.2 | 9.1 |  | - | - | 4.1 | 8.2 |  |
| Role Physical | 33.9 | 8.6 | 36.0 | 9.1 | 0.0035 | 34.7 | 9.0 | 36.5 | 9.1 | 0.012 | 34.2 | 8.8 | 38.0 | 9.9 | <0.0001 |
| Change from baseline, mean (SD) | - | - | 2.1 | 7.6 |  | - | - | 2.0 | 8.4 |  | - | - | 3.8 | 8.6 |  |
| Bodily Pain | 44.0 | 9.8 | 44.1 | 9.7 | 0.8612 | 44.5 | 10.0 | 45.1 | 10.5 | 0.5116 | 43.5 | 9.7 | 44.5 | 10.3 | 0.2501 |
| Change from baseline, mean (SD) | - | - | 0.1 | 6.6 |  | - | - | 0.5 | 8.7 |  | - | - | 0.9 | 7.8 |  |
| Vitality | 39.3 | 7.4 | 40.7 | 6.8 | 0.0414 | 40.8 | 7.4 | 41.9 | 7.9 | 0.1558 | 39.6 | 7.2 | 42.1 | 7.2 | 0.0015 |
| Change from baseline, mean (SD) | - | - | 1.4 | 7.3 |  | - | - | 1.0 | 7.7 |  | - | - | 2.4 | 7.3 |  |
| Social Functioning | 39.1 | 9.0 | 40.4 | 9.4 | 0.1348 | 39.5 | 9.0 | 41.5 | 9.6 | 0.0325 | 38.2 | 9.1 | 40.8 | 9.9 | 0.0116 |
| Change from baseline, mean (SD) | - | - | 1.3 | 9.2 |  | - | - | 2.1 | 10.4 |  | - | - | 2.6 | 10.0 |  |
| Mental Health | 43.6 | 9.6 | 44.7 | 9.8 | 0.0975 | 44.2 | 9.0 | 45.4 | 9.9 | 0.2635 | 43.5 | 9.7 | 45.9 | 10.3 | 0.004 |
| Change from baseline, mean (SD) | - | - | 1.2 | 7.6 |  | - | - | 1.0 | 9.6 |  | - | - | 2.4 | 8.0 |  |
| Role Emotional | 41.1 | 9.2 | 41.6 | 9.2 | 0.5356 | 41.4 | 9.4 | 42.4 | 9.5 | 0.2924 | 40.9 | 9.2 | 42.6 | 9.3 | 0.0838 |
| Change from baseline, mean (SD) | - | - | 0.5 | 8.1 |  | - | - | 1.0 | 10.0 |  | - | - | 1.7 | 9.8 |  |
| Physical Summary Score | 33.7 | 8.5 | 35.8 | 8.6 | 0.0011 | 34.4 | 8.8 | 36.3 | 9.4 | 0.0091 | 33.6 | 8.6 | 37.2 | 10.2 | <0.0001 |
| Change from baseline, mean (SD) | - | - | 2.1 | 7.0 |  | - | - | 2.1 | 8.3 |  | - | - | 3.5 | 8.0 |  |
| Mental Summary Score | 43.1 | 10.2 | 43.9 | 10.9 | 0.2618 | 44.0 | 9.9 | 45.0 | 11.2 | 0.2276 | 43.0 | 10.4 | 45.2 | 11.0 | 0.0074 |
| Change from baseline, mean (SD) | - | - | 0.8 | 8.0 |  | - | - | 1.3 | 10.8 |  | - | - | 2.5 | 8.9 |  |

Key: SD – standard deviation.

^a^The global score was calculated among patients who answered all the questions.

**Table S4. Number of Fainting Episodes, Good Days, and Bad Days**

| **Characteristic** | All Patients Contributing Month 1 Data | | | | | | All Patients Contributing Month 3 Data | | | | | | All Patients Contributing Month 6 Data | | | | | |
| --- | --- | --- | --- | --- | --- | --- | --- | --- | --- | --- | --- | --- | --- | --- | --- | --- | --- | --- |
|  | Baseline | | | 1 month | | *P*- value | Baseline | | | 3 month | | *P*- value | Baseline | | | 6 month | | *P*- value |
|  | N | Mean | SD | Mean | SD |  | N | Mean | SD | Mean | SD |  | N | Mean | SD | Mean | SD |  |
|  |  |  |  |  |  |  |  |  |  |  |  |  |  |  |  |  |  |  |
| Over the Past Week, How Many Times  Did You Lose Consciousness/Faint? | 121 | 0.6 | 1.6 | 0.5 | 1.4 | 0.3172 | 106 | 0.5 | 1.5 | 0.3 | 0.7 | 0.1583 | 94 | 0.4 | 1.3 | 0.4 | 1.5 | 0.8950 |
|  |  |  |  |  |  |  |  |  |  |  |  |  |  |  |  |  |  |  |
| Over the Past Week, How Many  Good Days Did You Have? | 130 | 3.1 | 2.0 | 4.0 | 2.1 | <0.0001 | 112 | 3.3 | 2.1 | 4.5 | 1.9 | <0.0001 | 97 | 3.3 | 2.1 | 4.5 | 2.1 | <0.0001 |
|  |  |  |  |  |  |  |  |  |  |  |  |  |  |  |  |  |  |  |
| Over the Past Week, How Many  Bad Days Did You Have? Mean (SD) | 128 | 3.7 | 2.1 | 2.9 | 2.0 | <0.0001 | 109 | 3.5 | 2.1 | 2.4 | 1.9 | <0.0001 | 95 | 3.5 | 2.1 | 2.4 | 2.0 | <0.0001 |

Key: SD – standard deviation

.

**Table S5. Patient Health Status: Patient Health Questionnaire-9 Scores**

| Characteristic | All Patients Contributing Month 1 Data | | | | | All Patients Contributing Month 3 Data | | | | | All Patients Contributing Month 6 Data | | | | |
| --- | --- | --- | --- | --- | --- | --- | --- | --- | --- | --- | --- | --- | --- | --- | --- |
|  | Baseline | | 1 month | | *P*-value | Baseline | | 3 month | | *P*-value | Baseline | | 6 month | | *P*-value |
|  | N | 115 | N | 115 |  | N | 115 | N | 115 |  | N | 101 | N | 101 |  |
| **PHQ-9 scores** |  |  |  |  |  |  |  |  |  |  |  |  |  |  |  |
| N | 115 |  | 115 |  |  | 102 |  |  |  |  | 91 |  |  |  |  |
| Score, mean (SD) | 11.0 | 6.3 | 9.7 | 6.3 | <0.01 | 10.6 | 6.4 | 8.9 | 6.3 | 0.01 | 11.2 | 6.2 | 8.6 | 6.1 | <0.01 |
| Change from baseline, mean (SD) | - | - | -1.3 | 4.5 | - | - | - | -1.7 | 6.2 | - | - | - | -2.6 | 5.1 | - |
| **PHQ-9 scores, n (%)** |  |  |  |  |  |  |  |  |  |  |  |  |  |  |  |
| Minimal | 20 | (17.4%) | 29 | (25.2%) | 0.01 | 22 | (19.1%) | 35 | (30.4%) | 0.03 | 15 | (14.9%) | 32 | (31.7%) | <0.01 |
| Mild | 36 | (31.3%) | 30 | (26.1%) |  | 30 | (26.1%) | 26 | (22.6%) |  | 28 | (27.7%) | 28 | (27.7%) |  |
| Moderate | 17 | (14.8%) | 29 | (25.2%) |  | 20 | (17.4%) | 20 | (17.4%) |  | 20 | (19.8%) | 17 | (16.8%) |  |
| Moderately severe | 33 | (28.7%) | 19 | (16.5%) |  | 27 | (23.5%) | 20 | (17.4%) |  | 23 | (22.8%) | 15 | (14.9%) |  |
| Severe | 9 | (7.8%) | 8 | (7.0%) |  | 7 | (6.1%) | 7 | (6.1%) |  | 8 | (7.9%) | 5 | (5.0%) |  |
| Missing/not complete | 0 | (0.0%) | 0 | (0.0%) |  | 9 | (7.8%) | 7 | (6.1%) |  | 7 | (6.9%) | 4 | (4.0%) |  |
| **Individual items** |  |  |  |  |  |  |  |  |  |  |  |  |  |  |  |
| Little interest or pleasure in doing things, n (%) |  |  |  |  |  |  |  |  |  |  |  |  |  |  |  |
| Not at all | 29 | (25.2%) | 33 | (28.7%) | 0.034 | 34 | (29.6%) | 40 | (34.8%) | 0.13 | 25 | (24.8%) | 39 | (38.6%) | <0.01 |
| Several days | 40 | (34.8%) | 42 | (36.5%) |  | 37 | (32.2%) | 33 | (28.7%) |  | 32 | (31.7%) | 31 | (30.7%) |  |
| More than half the days | 20 | (17.4%) | 27 | (23.5%) |  | 23 | (20.0%) | 32 | (27.8%) |  | 21 | (20.8%) | 20 | (19.8%) |  |
| Nearly every day | 26 | (22.6%) | 13 | (11.3%) |  | 20 | (17.4%) | 9 | (7.8%) |  | 23 | (22.8%) | 11 | (10.9%) |  |
| Unknown | 0 | (0.0%) | 0 | (0.0%) |  | 1 | (0.9%) | 1 | (0.9%) |  | 0 | (0.0%) | 0 | (0.0%) |  |
| Feeling down, depressed, or hopeless, n (%) |  |  |  |  |  |  |  |  |  |  |  |  |  |  |  |
| Not at all | 41 | (35.7%) | 47 | (40.9%) | 0.05 | 41 | (35.7%) | 49 | (42.6%) | 0.06 | 38 | (37.6%) | 43 | (42.6%) | 0.09 |
| Several days | 40 | (34.8%) | 39 | (33.9%) |  | 38 | (33.0%) | 35 | (30.4%) |  | 28 | (27.7%) | 31 | (30.7%) |  |
| More than half the days | 20 | (17.4%) | 18 | (15.7%) |  | 20 | (17.4%) | 21 | (18.3%) |  | 21 | (20.8%) | 15 | (14.9%) |  |
| Nearly every day | 14 | (12.2%) | 11 | (9.6%) |  | 14 | (12.2%) | 8 | (7.0%) |  | 13 | (12.9%) | 12 | (11.9%) |  |
| Unknown | 0 | (0.0%) | 0 | (0.0%) |  | 2 | (1.7%) | 2 | (1.7%) |  | 1 | (1.0%) | 0 | (0.0%) |  |
| Trouble falling asleep, staying asleep, or sleeping too much, n (%) |  |  |  |  |  |  |  |  |  |  |  |  |  |  |  |
| Not at all | 33 | (28.7%) | 32 | (27.8%) | 0.40 | 33 | (28.7%) | 31 | (27.0%) | 0.01 | 26 | (25.7%) | 36 | (35.6%) | <0.01 |
| Several days | 18 | (15.7%) | 27 | (23.5%) |  | 19 | (16.5%) | 39 | (33.9%) |  | 19 | (18.8%) | 29 | (28.7%) |  |
| More than half the days | 24 | (20.9%) | 19 | (16.5%) |  | 22 | (19.1%) | 16 | (13.9%) |  | 22 | (21.8%) | 13 | (12.9%) |  |
| Nearly every day | 40 | (34.8%) | 37 | (32.2%) |  | 40 | (34.8%) | 26 | (22.6%) |  | 34 | (33.7%) | 23 | (22.8%) |  |
| Unknown | 0 | (0.0%) | 0 | (0.0%) |  | 1 | (0.9%) | 3 | (2.6%) |  | 0 | (0.0%) | 0 | (0.0%) |  |
| Feeling tired or having little energy, n (%) |  |  |  |  |  |  |  |  |  |  |  |  |  |  |  |
| Not at all | 4 | (3.5%) | 7 | (6.1%) | 0.01 | 8 | (7.0%) | 13 | (11.3%) | <0.01 | 6 | (5.9%) | 13 | (12.9%) | <0.01 |
| Several days | 25 | (21.7%) | 35 | (30.4%) |  | 27 | (23.5%) | 37 | (32.2%) |  | 17 | (16.8%) | 32 | (31.7%) |  |
| More than half the days | 25 | (21.7%) | 23 | (20.0%) |  | 25 | (21.7%) | 25 | (21.7%) |  | 23 | (22.8%) | 23 | (22.8%) |  |
| Nearly every day | 61 | (53.0%) | 50 | (43.5%) |  | 54 | (47.0%) | 38 | (33.0%) |  | 55 | (54.5%) | 33 | (32.7%) |  |
| Unknown | 0 | (0.0%) | 0 | (0.0%) |  | 1 | (0.9%) | 2 | (1.7%) |  | 0 | (0.0%) | 0 | (0.0%) |  |
| Poor appetite or overeating, n (%) |  |  |  |  |  |  |  |  |  |  |  |  |  |  |  |
| Not at all | 39 | (33.9%) | 44 | (38.3%) | 0.25 | 43 | (37.4%) | 49 | (42.6%) | 0.17 | 36 | (35.6%) | 44 | (43.6%) | 0.17 |
| Several days | 34 | (29.6%) | 33 | (28.7%) |  | 26 | (22.6%) | 28 | (24.3%) |  | 28 | (27.7%) | 25 | (24.8%) |  |
| More than half the days | 21 | (18.3%) | 20 | (17.4%) |  | 19 | (16.5%) | 17 | (14.8%) |  | 15 | (14.9%) | 17 | (16.8%) |  |
| Nearly every day | 21 | (18.3%) | 18 | (15.7%) |  | 22 | (19.1%) | 18 | (15.7%) |  | 19 | (18.8%) | 14 | (13.9%) |  |
| Unknown | 0 | (0.0%) | 0 | (0.0%) |  | 5 | (4.3%) | 3 | (2.6%) |  | 3 | (3.0%) | 1 | (1.0%) |  |
| Feeling bad about yourself, n (%) |  |  |  |  |  |  |  |  |  |  |  |  |  |  |  |
| Not at all | 55 | (47.8%) | 52 | (45.2%) | 0.47 | 52 | (45.2%) | 64 | (55.7%) | 0.01 | 49 | (48.5%) | 54 | (53.5%) | 0.13 |
| Several days | 26 | (22.6%) | 39 | (33.9%) |  | 29 | (25.2%) | 29 | (25.2%) |  | 19 | (18.8%) | 26 | (25.7%) |  |
| More than half the days | 16 | (13.9%) | 14 | (12.2%) |  | 17 | (14.8%) | 13 | (11.3%) |  | 14 | (13.9%) | 6 | (5.9%) |  |
| Nearly every day | 18 | (15.7%) | 10 | (8.7%) |  | 14 | (12.2%) | 8 | (7.0%) |  | 16 | (15.8%) | 13 | (12.9%) |  |
| Unknown | 0 | (0.0%) | 0 | (0.0%) |  | 3 | (2.6%) | 1 | (0.9%) |  | 3 | (3.0%) | 2 | (2.0%) |  |
| Trouble concentrating on things, n (%) |  |  |  |  |  |  |  |  |  |  |  |  |  |  |  |
| Not at all | 42 | (36.5%) | 44 | (38.3%) | 0.30 | 45 | (39.1%) | 51 | (44.3%) | 0.06 | 38 | (37.6%) | 46 | (45.5%) | 0.08 |
| Several days | 32 | (27.8%) | 38 | (33.0%) |  | 31 | (27.0%) | 35 | (30.4%) |  | 28 | (27.7%) | 27 | (26.7%) |  |
| More than half the days | 23 | (20.0%) | 17 | (14.8%) |  | 21 | (18.3%) | 16 | (13.9%) |  | 22 | (21.8%) | 16 | (15.8%) |  |
| Nearly every day | 18 | (15.7%) | 16 | (13.9%) |  | 17 | (14.8%) | 12 | (10.4%) |  | 13 | (12.9%) | 12 | (11.9%) |  |
| Unknown | 0 | (0.0%) | 0 | (0.0%) |  | 1 | (0.9%) | 1 | (0.9%) |  | 0 | (0.0%) | 0 | (0.0%) |  |
| Moving or speaking so slowly that others could have noticed, n (%) |  |  |  |  |  |  |  |  |  |  |  |  |  |  |  |
| Not at all | 48 | (41.7%) | 50 | (43.5%) | 0.01 | 52 | (45.2%) | 59 | (51.3%) | 0.01 | 41 | (40.6%) | 49 | (48.5%) | 0.01 |
| Several days | 20 | (17.4%) | 34 | (29.6%) |  | 21 | (18.3%) | 26 | (22.6%) |  | 18 | (17.8%) | 25 | (24.8%) |  |
| More than half the days | 28 | (24.3%) | 21 | (18.3%) |  | 23 | (20.0%) | 20 | (17.4%) |  | 23 | (22.8%) | 13 | (12.9%) |  |
| Nearly every day | 19 | (16.5%) | 10 | (8.7%) |  | 18 | (15.7%) | 8 | (7.0%) |  | 19 | (18.8%) | 13 | (12.9%) |  |
| Unknown | 0 | (0.0%) | 0 | (0.0%) |  | 1 | (0.9%) | 2 | (1.7%) |  | 0 | (0.0%) | 1 | (1.0%) |  |
| Thoughts that you would be better off dead or of hurting yourself in some way, n (%) |  |  |  |  |  |  |  |  |  |  |  |  |  |  |  |
| Not at all | 94 | (81.7%) | 100 | (87.0%) | 0.11 | 94 | (81.7%) | 96 | (83.5%) | 0.69 | 83 | (82.2%) | 87 | (86.1%) | 0.23 |
| Several days | 17 | (14.8%) | 10 | (8.7%) |  | 12 | (10.4%) | 14 | (12.2%) |  | 13 | (12.9%) | 11 | (10.9%) |  |
| More than half the days | 3 | (2.6%) | 5 | (4.3%) |  | 4 | (3.5%) | 2 | (1.7%) |  | 3 | (3.0%) | 1 | (1.0%) |  |
| Nearly every day | 1 | (0.9%) | 0 | (0.0%) |  | 3 | (2.6%) | 2 | (1.7%) |  | 2 | (2.0%) | 2 | (2.0%) |  |
| Unknown | 0 | (0.0%) | 0 | (0.0%) |  | 2 | (1.7%) | 1 | (0.9%) |  | 0 | (0.0%) | 0 | (0.0%) |  |
| If you checked off any problems, how difficult have those problems made it for you to do your work, take care of things at home, or get along with other people, n (%) |  |  |  |  |  |  |  |  |  |  |  |  |  |  |  |
| Not difficult at all | 21 | (18.3%) | 24 | (20.9%) | 0.16 | 25 | (21.7%) | 36 | (31.3%) | 0.05 | 15 | (14.9%) | 25 | (24.8%) | 0.02 |
| Somewhat difficult | 56 | (48.7%) | 54 | (47.0%) |  | 52 | (45.2%) | 47 | (40.9%) |  | 53 | (52.5%) | 51 | (50.5%) |  |
| Very difficult | 18 | (15.7%) | 26 | (22.6%) |  | 23 | (20.0%) | 20 | (17.4%) |  | 18 | (17.8%) | 16 | (15.8%) |  |
| Extremely difficult | 19 | (16.5%) | 8 | (7.0%) |  | 14 | (12.2%) | 7 | (6.1%) |  | 14 | (13.9%) | 7 | (6.9%) |  |
| Unknown | 1 | (0.9%) | 3 | (2.6%) |  | 1 | (0.9%) | 5 | (4.3%) |  | 1 | (1.0%) | 2 | (2.0%) |  |

Key: SD – standard deviation.

**(4) METHODS**

***Study Design***

This was a non-interventional, United States-based, prospective cohort study in patients newly initiating droxidopa for the treatment of nOH. Data for this study were reported by study participants and the HUB. Data were collected using case report forms, which were developed in collaboration with 3 people who had nOH. Patients participated by completing the online, paper, or telephone interview assessment at 5 time points during the 6-month follow-up (screening, baseline, Month 1, Month 3, and Month 6). For patients who chose to participate via telephone, interviews were mailed and/or emailed in advance to facilitate the telephone-based completion of the assessments.

Patients newly prescribed droxidopa were provided a brief description of the study and asked about their interest in participation by HUB pharmacy personnel. Interested patients were referred to the study central intake center to determine eligibility during a telephone screening interview. Upon meeting study criteria, patients provided verbal consent on the telephone to trained study personnel. Patients were reimbursed for participation. Before study initiation and patient enrollment, the protocol and informed consent form were submitted to Schulman Institutional Review Board, a central Institutional Review Board, for study approval. This study was conducted in accordance with the protocol and was consistent with The International Council for Harmonisation Standards of Good Clinical Practice and the applicable regulatory requirements.

***Statistical Analyses for LTFU***

LTFU analysis was conducted to examine potential bias in findings related to falling and PROs. The association between LTFU at Month 6 and select baseline characteristics and baseline outcomes were also assessed. This assessment determined whether LTFU was random, (i.e., if LTFU was not associated with the baseline outcome). For instance, if those patients reporting a fall at baseline were more likely to drop out of the study, then LTFU would not be random and the study results would be biased. Therefore, a multivariable logistic regression model was fit where the outcome measure was a binary variable for LTFU at Month 6 and the main independent variable was the baseline measure of the outcome (fall, OHSA-1, FES-1, SDS, SF-8, and PHQ-9). Separate models were generated for assessment of the impact of each baseline measure of the outcome on LTFU at Month 6. Additional model covariates were age, gender, primary diagnosis, midodrine, fludrocortisone, living status, and droxidopa treatment status at Month 6. A significant coefficient for the baseline measure of outcome was considered to be evidence of potential bias due to LTFU for that outcome.
